# Effect of an educational intervention for telephone triage nurses on out-of-hours attendance: a pragmatic randomized controlled study

**DOI:** 10.1186/s12913-022-08994-0

**Published:** 2023-01-03

**Authors:** Bent Håkan Lindberg, Ingrid Keilegavlen Rebnord, Sigurd Høye

**Affiliations:** 1 Antibiotic Centre for Primary Care, Department of General Practice, Institute of Health and Society, University of Oslo, 0315 Oslo, Norway; 2 National Centre for Emergency Primary Health Care, NORCE Norwegian Research Centre, Bergen, Norway; 3 Department of Global Public Health and Primary Care, University of Bergen, Bergen, Norway

**Keywords:** Out-of-hours, Telephone triage, Nurse, Educational intervention, Respiratory tract infections, Primary health care

## Abstract

**Background:**

Telephone triage has been established in many countries as a response to the challenge of non-urgent use of out-of-hours primary care services. However, limited evidence is available regarding the effect of training interventions on clinicians’ telephone consultation skills and patient outcomes.

**Methods:**

This was a pragmatic randomized controlled educational intervention for telephone triage nurses in 59 Norwegian out-of-hours general practitioners’ (GPs) cooperatives, serving 59% of the Norwegian population. Computer-generated randomization was performed at the level of out-of-hours GP cooperatives, stratified by the population size. Thirty-two out-of-hours GP cooperatives were randomized to intervention. One cooperative did not accept the invitation to participate in the educational programme, leaving 31 cooperatives in the intervention group. The intervention comprised a 90-minute e-learning course and 90-minute group discussion about respiratory tract infections (RTIs), telephone communication skills and local practices.

We aimed to assess the effect of the intervention on out-of-hours attendance and describe the distribution of RTIs between out-of-hours GP cooperatives and list-holding GPs.

The outcome was the difference in the number of doctor’s consultations per 1000 inhabitants between the intervention and control groups during the winter months before and after the intervention. A negative binomial regression model was used for the statistical analyses. The model was adjusted for the number of nurses who had participated in the e-learning course, the population size and patients’ age groups, with the out-of-hours GP cooperatives defined as clusters.

**Results:**

The regression showed that the intervention did not change the number of consultations for RTIs between the two groups of out-of-hours GP cooperatives (incidence rate ratio 0.99, 95% confidence interval 0.91–1.07). The winter season’s out-of-hours patient population was younger and had a higher proportion of RTIs than the patient population in the list-holding GP offices. Laryngitis, sore throat, and pneumonia were the most common diagnoses during the out-of-hours primary care service.

**Conclusions:**

The intervention did not influence the out-of-hours attendance. This finding may be due to the intervention’s limited scope and the intention-to-treat design. Changing a population’s out-of-hours attendance is complicated and needs to be targeted at several organizational levels.

**Supplementary Information:**

The online version contains supplementary material available at 10.1186/s12913-022-08994-0.

## Background

Out-of-hours primary care service is a crucial part of a well-functioning health care system, which provides health care when general practitioners’ (GPs) offices are closed. The member countries of the Organisation for Economic Co-operation and Development have selected different models to provide this kind of service to their populations [1]. GP cooperatives are the dominant model used in Europe [2].

The non-urgent use of out-of-hours service, both in primary care and emergency department setting, is of concern, because it may crowd out patients with the highest need and lead to inefficient use of resources [3–8]. Telephone triage has been implemented in many countries as a response to the challenge of non-urgent use of out-of-hours service. However, evidence regarding the effect of this measure on the use of out-of-hours service is limited [7]. Nevertheless, most European countries have implemented or improved the quality of telephone triage during the last 10 years [2].

Some countries, such as the UK, have a national telephone triage and advice system. Norway has one national telephone number for out-of-hours service; however, nurses in local out-of-hours GP cooperatives handle the calls from the local population. A review from 2017 suggested that more than 50% of calls to the telephone triage and advice system could be dealt with by providing telephone advice alone [9]. However, only 23% of incoming calls to the Norwegian out-of-hours service are managed by nurses’ telephone counselling alone [10].

High variation has been reported in measured appropriateness of advice provided by the telephone triage and advice services [9]. High quality of nurses’ telephone consultations positively affects the appropriateness of triage decisions, and a positive correlation exists between the duration of nurses’ telephone consultations and the overall communication score [11, 12]. Patient-centred communication, active listening and advising, as well as structured calls, may improve the quality of telephone triage [12].

Overall, telephone triage in out-of-hours care appears to be safe because high-risk patients are mostly identified and handled correctly [13]. However, limited evidence is available regarding the triage of callers with mild-to-moderate symptoms, such as self-limiting acute respiratory tract infections (RTIs). These conditions are non-urgent and do not necessarily require medical attention out-of-hours, even in a primary care setting. They increase the workload and busyness in the out-of-hours care, which in turn may impair clinical quality of care, as demonstrated in the case of antibiotic prescription for RTIs [14]. Telephone triage has the potential to reduce the workload of GPs [9]. Nevertheless, telephone triage nurses are reluctant to call themselves gatekeepers to the out-of-hours GP cooperatives [15]. They rather describe themselves as service providers who want to reach a consensus with the caller about the right level of care. They argue that such agreement is easier to reach if they have proper knowledge of the relevant guidelines. Consequently, a gap exists between the needs and expectations of stakeholders of primary health care as well as the needs of and reality experienced by telephone triage nurses.

Norwegian citizens are entitled to have a list-holding GP who offer planned and emergent service for their listed patients in-hour [16]. The list-holding GPs are also obliged to work sessions in the out-of-hours service. Yet, other doctors such as locums and hospital doctors manage 42% of the out-of-hours consultations [17]. The municipalities organize their out-of-hours service according to local needs and geographical variations, alone or together with neighbouring municipalities. Forty-nine % of the services cover more than one municipality [18]. The median population size per out-of-hours service was 12,823 (450–699,827) in 2022 [19]. Out-of-hours GP cooperative (with more than 15 doctors taking turns being on duty) is the most common model for organizing out-of-hours service, but rota groups (four to fifteen doctors) and individual GP practices (less than four doctors) are also common [2]. A nurse or paramedic with a bachelor’s degree is required to answer all incoming calls to the local out-of-hours service, and at least one medical doctor must be available for physical consultations 24/7 [20]. There is no record on the number of doctors on duty, but the median number of nurses employed per out-of-hours service was 17.5 in 2018, with large variation (1- > 50) [21]. In most services the nurses rotate between answering the telephone from the public and supporting the doctor in the clinical work. Primary care physicians, i.e., list holding GPs and other out-of-hours doctors, are mandatory gatekeepers to secondary health care 24/7. Patients pay a medical fee per consultation, which is 75% higher out-of-hour than in-hour. When the maximum amount of 2921 NOK (295 Euros) per year is paid, any further consultations in the current year are free of charge.

High primary care physician continuity leads to lower costs in health care, fewer hospitalizations, and lower mortality [22, 23]. Hence, one can argue that callers who can wait for the list-holding GP’s opening hours should wait, including those with RTIs. A long-lasting doctor–patient relationship is a better basis for practising a restrained antibiotic prescribing attitude than a busy out-of-hours session [14]. A transfer of 20% of patients from Norwegian out-of-hours service to day-time practice would lead to a 1.6% increase in the number of consultations for list-holding GPs [24, 25]. Despite work overload and recruitment problems in the Norwegian regular GP scheme, we believe that this increase would be manageable and pertinent [26].

Specific training may strengthen telephone triage nurses’ gatekeeping capacity. However, insufficient evidence is available to conclude the effectiveness of interventions aimed at reducing emergency department attendance [27]. Existing evidence is characterized by heterogeneous patient groups, unspecific target groups and the lack of control groups [27]. A Cochrane report from 2017 concluded that more research assessing the effect of different training interventions on clinicians’ telephone consultation skills and their effect on patient outcomes is needed [28].

The primary aim of this study was to assess the effect of an educational intervention about RTIs and communication skills for telephone triage nurses on out-of-hours attendance for RTIs. The secondary aims were to describe the distribution of RTIs between list-holding GPs and out-of-hours GP cooperatives and assess the intervention’s effect on list-holding GPs’ attendance.

## Methods

### Study design

This is a pragmatic randomized controlled educational intervention among Norwegian telephone triage nurses working in out-of-hours GP cooperatives. All Norwegian out-of-hours GP cooperatives that met our inclusion criteria were included in the study (Table 1).

**Table 1:** Inclusion criteria for out-of-hours GP cooperatives

1. Serving a population > 10,000 inhabitants	
2. Advising the population to call the service on perceiving a need for medical attention out-of-hours	
3. The telephone office answering the national telephone number for the out-of-hours service is localized inside the out-of-hours GP cooperative	
4. Is managed by at least one nurse during evenings	
5. Covers one or several entire municipalities (municipalities where the population adheres to only one out-of-hours GP cooperative)	

The out-of-hours services in the two largest cities of Norway, Bergen, and Oslo, were not included owing to their policy of direct attendance. Out-of-hours cooperatives serving less than 10,000 inhabitants were excluded as they normally will have less than 6 consultations per 24 hours and therefore are less busy [24]. Also, these cooperatives seldom have conflicts of simultaneity and are therefore less likely to use triage scales [18].

A total of 64 out-of-hours GP cooperatives serving 3.4 million inhabitants (median population size per out-of-hours GP cooperative 36.048, range 11.490–236.202) met the inclusion criteria and were randomized to intervention or control. An independent researcher performed the randomization using the RAND command in Microsoft Excel (2016) in November 2018, with stratification by the population size (small 10,000–40,000; large > 40,001).

**Fig. 1:**
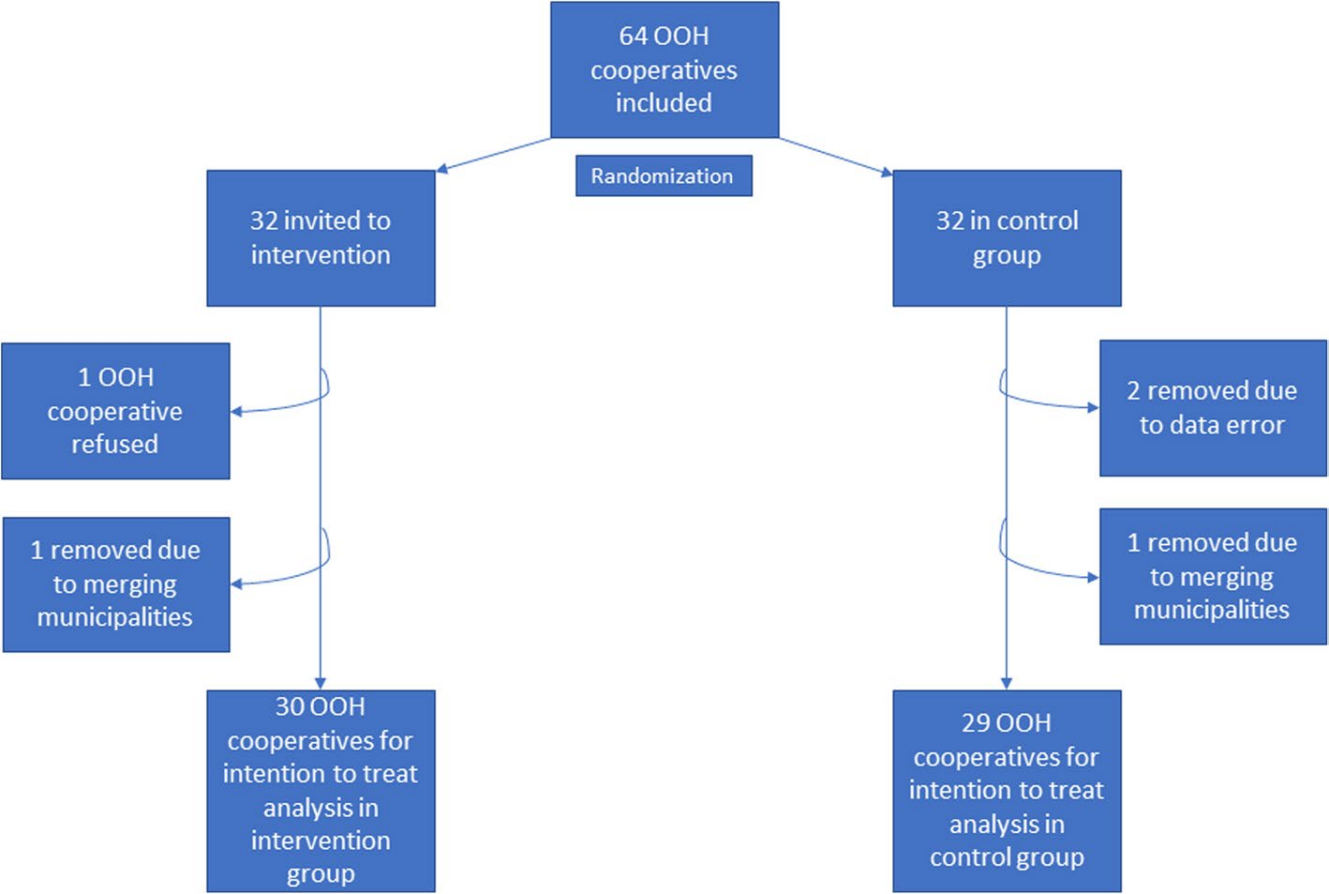
Flow chart of out-of-hours cooperatives included and reasons for exclusion after randomization

We contacted the administration of the 32 out-of-hours cooperatives in the intervention group through e-mail or telephone and invited them to participate in a meeting during the annual leadership conference for the primary care out-of-hours service in Norway (Lederkonferansen 2019) in March 2019. In this meeting, we presented the educational intervention and explained how they could implement it locally without any external support. Nineteen persons from 16 out-of-hours GP cooperatives participated. BHL contacted the other 16 out-of-hours GP cooperatives, three by personal contact during the conference and the remaining via telephone calls after the conference. One of the 32 cooperatives refused to participate due to time constraint, while the other 31 accepted the invitation and were included in the study. Data from one out-of-hours cooperative in the intervention group, in addition to one out-of-hours cooperative and one municipality in the control group, were excluded from the study because of the merging of municipalities across the two groups during the study period. Data from two out-of-hours cooperatives (serving 180,989 inhabitants) in the control group were excluded because of data error. As a result, 59 out-of-hours GP cooperatives serving 59% of the Norwegian population, were included in the intention-to-treat analysis (Fig. 1).

### The intervention

The intervention consisted of two parts:

1. A 90 minute e-learning course about acute RTIs and general communication techniques for telephone triage nurses (see Appendix A for the outline). The authors prepared the course based on preliminary data from a qualitative study on telephone triage nurses’ assessment of RTIs [15].
2. A 90 minute group discussion for the telephone triage nurses. Dedicated local nurses led the group sessions based on a written guide (Appendix B). The main elements were to discuss gained knowledge from the e-learning course and to discuss the unique activity reports for each out-of-hours GP cooperative (Appendix C). The reports described how the population attended each out-of-hours GP cooperative and the respective GP offices during 2018, with emphasis on acute RTIs. The guide and reports were prepared by BHL and SH and were based on data from Statistics Norway and aggregated data from the open database of the Norwegian Control and Payment of Health Reimbursement registry.^1^

The intervention was piloted by ten telephone triage nurses at one out-of-hours GP cooperative in May 2019 and adjusted based on the feedback from these nurses.

The unique activity reports (Appendix C) were sent to the intervention cooperatives by mail and e-mail in September 2019. The e-learning course was launched password-protected on a web platform in October 2019, exclusively for the intervention cooperatives [29]. We encouraged the local contacts to implement both parts of the intervention before December 2019. The pragmatic intention-to-treat design implied no follow-up of the implementation. However, through the log-on function of the web platform, we could see how many nurses had participated in the e-learning course.

### Variables

GP offices and out-of-hours GP cooperatives send electronic compensation claims to the Norwegian Health Economics Administration. The claims contain data on date and type of contact (phone, consultation, or home visit) with GP offices and out-of-hours GP cooperatives as well as age, gender, and diagnosis of the caller/patient. We retrieved these data from the National Directory of Health [30].

We did not have access to the number of nurses working in each out-of-hours cooperative during the intervention. The number of nurses who had just started or completed the e-learning course per 1000 inhabitants in each region was therefore used as a proxy for the proportion of trained nurses in each out-of-hours GP cooperative.

The primary outcome was the change in the number of out-of-hours doctor’s consultations for RTIs per 1000 inhabitants in the intervention group versus the control group during the winter months before (December 2018–February 2019) and after (December 2019–February 2020) the intervention. The secondary outcomes per 1000 inhabitants during the same two periods were:

- the change in the number of list-holding GPs’ consultations for all diagnoses
- the change in the number of list-holding GPs’ consultations for RTIS
- the change in the number of telephone contacts for all diagnoses for list-holding GPs
- the change in the number of nurses’ telephone consultations for all diagnoses in the out-of-hours GP cooperatives

We defined RTIs according to the International Classification of Primary Care, 2nd edition, as the following groups: ‘respiratory symptoms’ (R01–05, R08–09, R21 and R23–29), acute tonsillitis (R72 and 76), acute RTIs (R74), acute sinusitis (R75), acute laryngitis (R77), acute bronchitis (R78), influenza infection (R80), pneumonia (R81–82), ‘other RTIs’ (R71, R83 and R99) and ear infections (H01, H29, H70–72 and H74). We also defined the groups urinary tract infections (U01–02, U07, U13 and U70–72), ‘other conditions’ (A03, A76–78), ‘unspecified’ (A99) and ‘all other diagnoses’ (all International Classification of Primary Care codes not included in any of the above-mentioned groups).

### Statistical analysis

We used frequencies and percentages to describe the distribution of the sample population and count data relating to contacts and consultations made in the control and intervention arms, and StataSE 17 (StataCorp. 2021. Stata Statistical Software: Release 17. College Station, TX: StataCorp LLC) for the statistical analyses.

The Poisson regression is the basic model used for modelling count data. It assumes that the mean and the variance of the count variable are approximately equal. We checked this model assumption using the dispersion statistic, which should be equal to one. However, our Poisson model output gave the dispersion statistic equal to 24.2. In addition, the appropriateness of the negative binomial model over the Poisson regression model was checked using the Bayesian information criterion (BIC), which states that the model with the smaller BIC estimate is preferred over the model with a larger BIC. The negative binomial model had the smaller BIC estimate. Thirdly, we obtained a Z score test of 16.1, with a t-probability < 0.01. This test evaluates whether the data are Poisson or negative binomial. Based on this result, we rejected the null hypothesis of no overdispersion in the data. Further, we used the likelihood-ratio test of alpha = 0, to test if the dispersion parameter, was equal to zero, which would have reduced the model to a Poisson regression. Our results showed that alpha was significantly greater than zero. This implies that the data were over-dispersed, hence using the negative binomial model instead of the Poisson model was appropriate.

The negative binomial regression model was used with random effects at the level of out-of-hours GP cooperatives to check for any baseline differences between the two groups (Table 2) and for the primary (Table 3) and secondary outcomes. The model was adjusted for the number of nurses who had only started or completed the e-learning course (per 1000 inhabitants), the population size and patients’ age groups, with the out-of-hours GP cooperatives defined as clusters. We also run a three-way interaction model as a sensitivity analysis for the variable of the number of nurses who had started or completed the e-learning course (per 1000 inhabitants).

We obtained estimates of incidence rate ratios (IRRs) for the negative binomial regression model. An IRR > 1 indicates an increase in counts in one group relative to the reference group or one period relative to the reference period, whereas an IRR < 1 indicates a decrease in these parameters. An IRR = 1 indicates no difference in the number of counts between the groups or periods.

## Results

### Population and epidemiology at baseline

The 59 out-of-hours cooperatives served 3.12 million inhabitants (59% of the Norwegian population) in 198 (of 356) municipalities. There were 4.72 million contacts in primary health care in these municipalities in the baseline period, of which 4.37 million were list-holding GPs’ consultations and 0.35 million were GP consultations in the out-of-hours service.

**Fig. 2:**
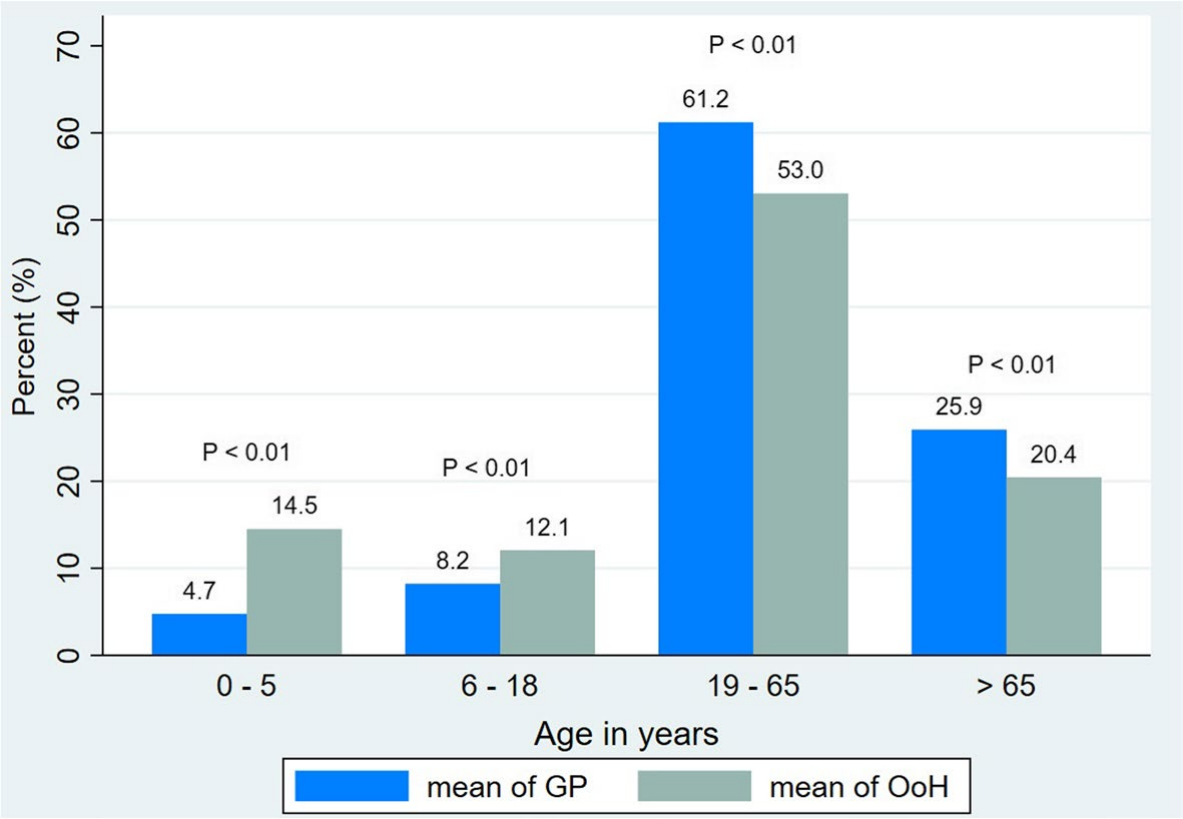
Proportion of age groups in GP offices and out-of-hours GP cooperatives, all diagnoses. *P*-values obtained from Chi-square test

Women constituted 52.5 and 57.8% of the patients who received service in out-of-hours cooperatives and GP offices, respectively. Patients were relatively younger in the out-of-hours cooperatives than in the GP offices (Fig. 2).

**Fig. 3:**
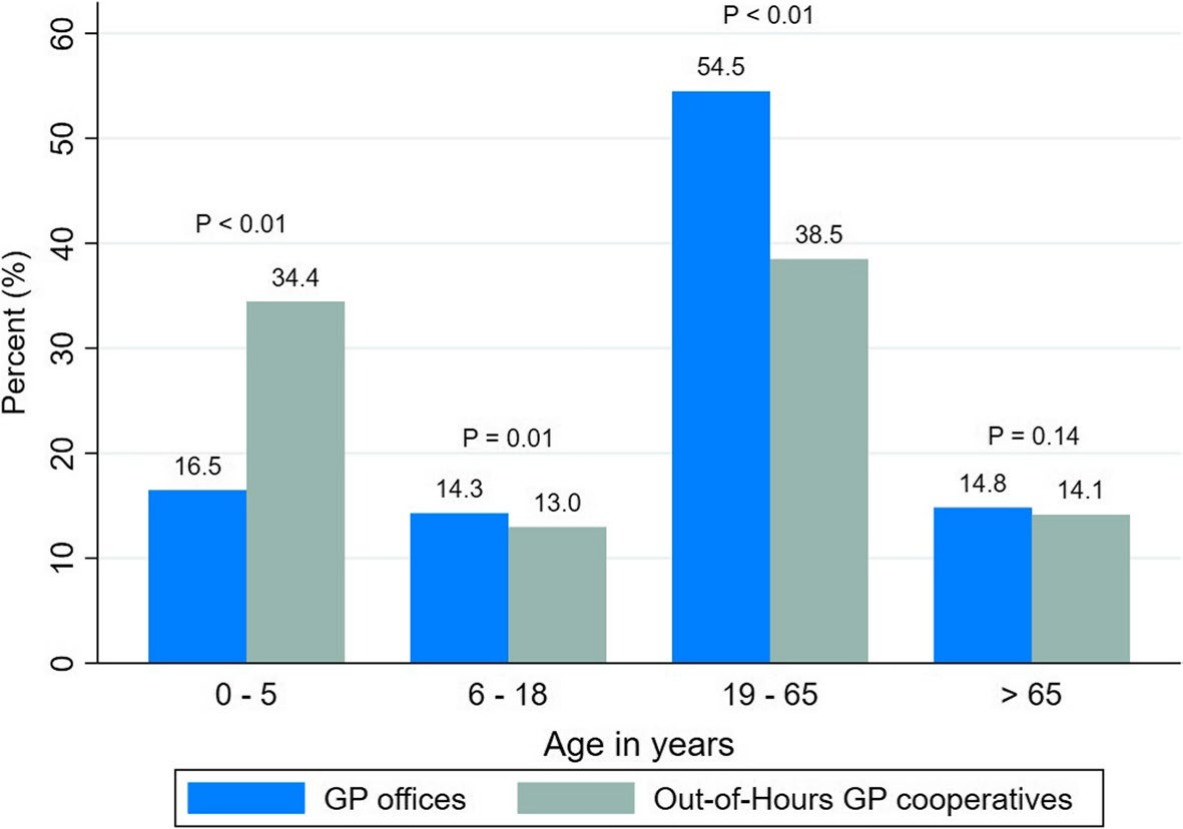
Proportion of age groups in GP offices and out-of-hours GP cooperatives, RTIs. *P*-values obtained from Chi-square test

For RTIs, 34.4 and 16.5% of children under 5 years of age received a diagnosis in out-of-hours cooperatives and GP offices, respectively (Fig. 3).

**Fig. 4:**
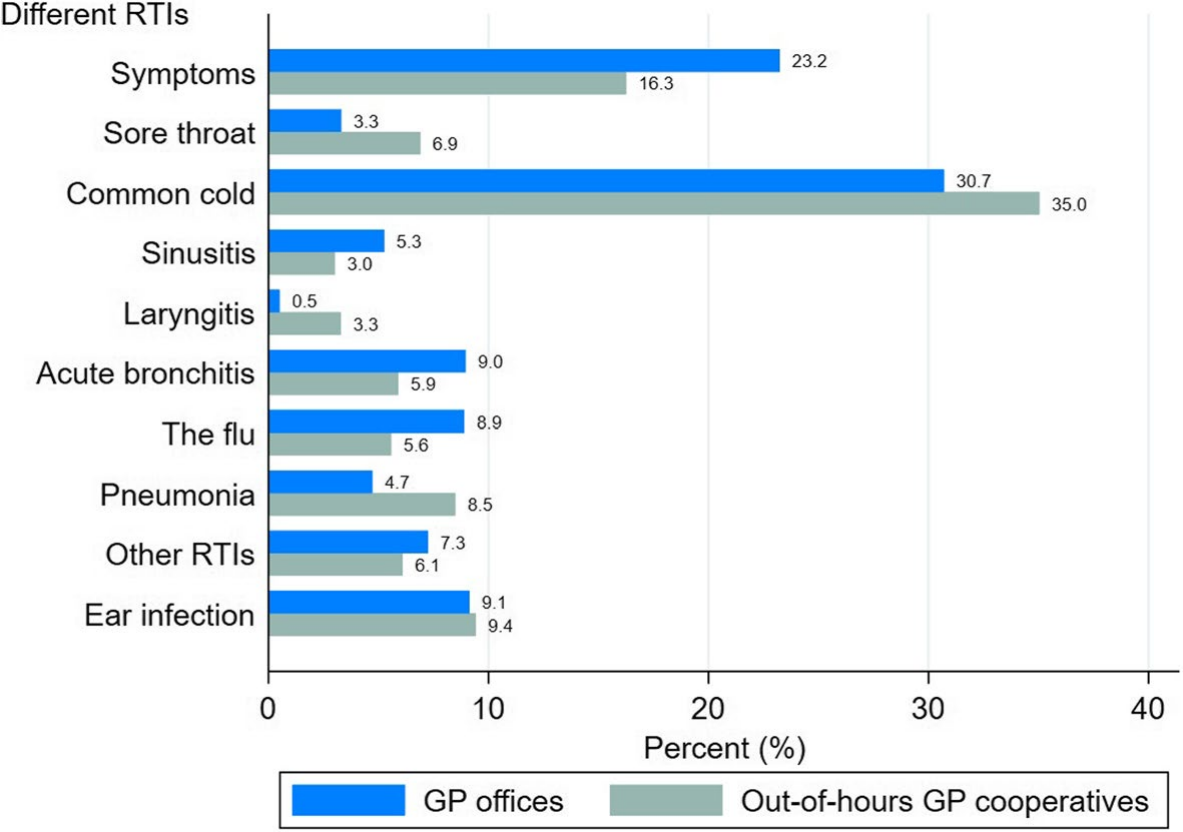
Proportion of different RTIs in GP offices and out-of-hours GP cooperatives. All the differences between GP offices and out-of-hours GP cooperatives, except for ear infections, were statistically significant (*p* < 0.05, Chi square test)

Respiratory symptoms and acute upper RTI were the two most frequently diagnosed RTIs (Fig. 4), and 13.9% of all RTIs were diagnosed in out-of-hours cooperatives.

**Fig. 5:**
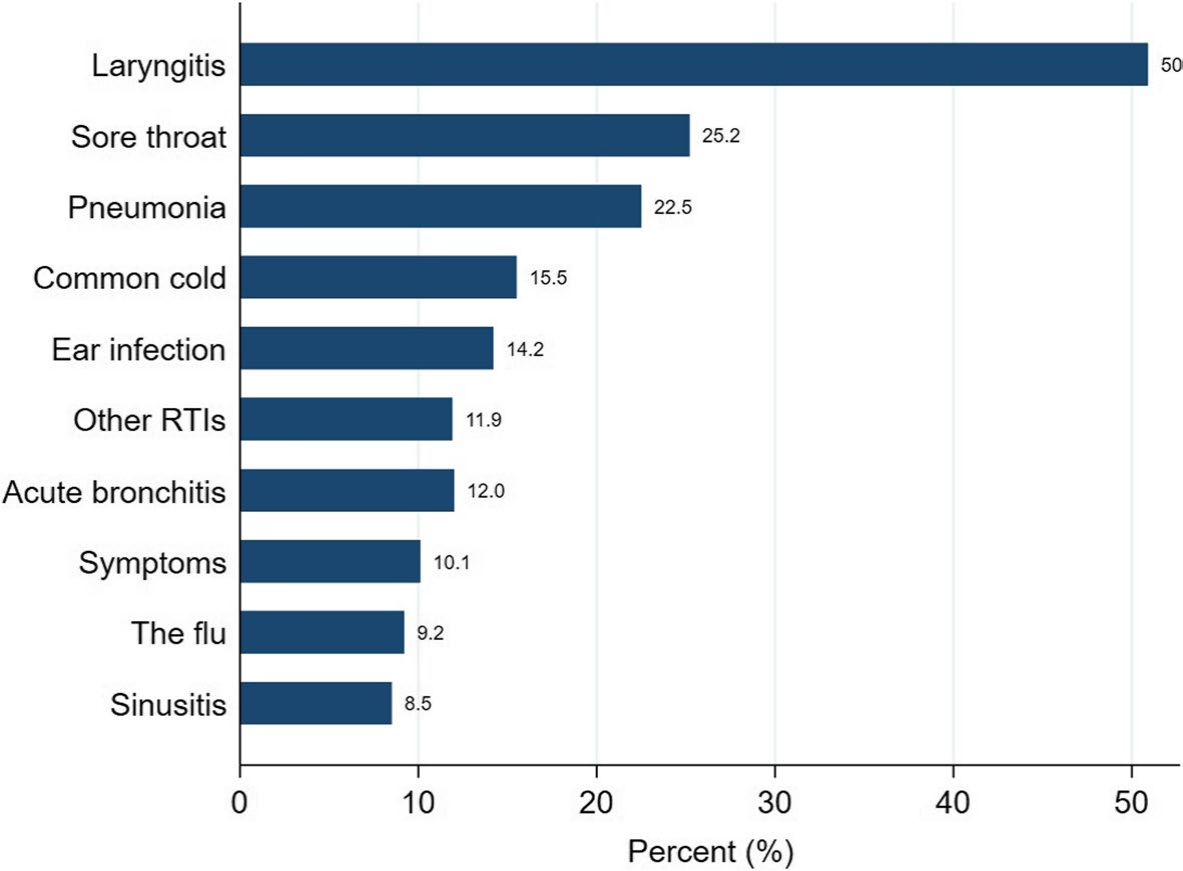
Proportion of conditions diagnosed in out-of-hours GP cooperatives

The proportion of RTIs diagnosed was higher in out-of-hours cooperatives than in the GP offices (22.9% versus 14.0%). Laryngitis, other conditions (viral RTIs frequently diagnosed in children) and sore throat were the most common diagnoses in the out-of-hours GP cooperatives (Fig. 5).

### Effect of the intervention

At baseline, no significant difference was observed between the intervention and control groups with regard to how the out-of-hours GP cooperatives and the list-holding GPs were used by the population (Table 2).

**Table 2:** Characteristics of the groups and adjusted estimates of IRR and their 95% CIs obtained from the negative binomial regression model showing the differences in counts between the groups at baseline

	Control	Per 1000 inhabitants	Intervention	Per 1000 inhabitants	IRR (95% CI, ref. control)
Number of out-of-hours GP cooperatives	29		30		
Population	1,557,545		1,561,723		
Total number of all contacts for out-of-hours cooperatives and list-holding GPs	2,361,584	1516	2,361,666	1512	0.97 (0.92–1.03)
Number of consultations for all diagnoses for out-of-hours cooperatives	103,772	67	109,859	70	0.94 (0.83–1.06)
Number of RTI consultations for out-of-hours cooperatives	23,589	15	25,321	16	0.97 (0.82–1.13)
Number of consultations for all diagnoses for list-holding GPs	1,077,563	692	1,098,182	703	0.99 (0.94–1.05)
Number of RTI consultations for list-holding GPs	152,536	98	151,179	97	1.04 (0.96–1.13)

*CI* confidence interval *GP* general physician*; IRR* incidence rate ratio*; RTI* respiratory tract infection*.* The model was adjusted for the number of nurses participating in the e-learning course, population size and patients’ age groups, with the out-of-hours GP cooperatives defined as clusters

By the end of November 2019, 286 nurses (61% of registered participating nurses) had completed the e-learning course. The negative binomial regression showed that the intervention led to no significant difference in the number of consultations for RTIs between the out-of-hours GP cooperatives in the intervention and control groups (IRR 0.99, 95% CI 0.91–1.07; Table 3).

**Table 3:** Adjusted estimates of IRR and their 95% CIs obtained from the negative binomial regression model showing the differences in counts between the groups before and after the intervention

		Before	After
Number of RTI consultations in the out-of-hours GP cooperatives	Control	23,589	22,956
	Intervention	25,321	25,245
	Difference	1732	2289
	IRR (95% CI, ref. control)	0.97 (0.82–1.13)	0.95 (0.82–1.11)
Difference in number of RTI consultations between the two groups after the intervention, ref. control	Numbers of consultations (ref. control)		557
	IRR (95% CI, ref. control)		0.99 (0.91–1.07)

*CI, confidence interval; IRR, incidence rate ratio; RTI, respiratory tract infection*

*The model was adjusted for the number of nurses participating in the e-learning course, population size and patients’ age groups, with the out-of-hours GP cooperatives defined as clusters. The model indicates that there is no difference in the number of RTI consultations between the two groups after the intervention*

The intervention did neither result in significant differences with regard to the number of telephone consultations for RTIs for out-of-hours GP cooperatives (IRR 0.83, 95% CI 0.66–1.07), the number of list-holding GPs’ consultations for all diagnoses (IRR 0.98, 95% CI 0.95–1.01) or RTIs (1.00, 95% CI 0.94–1.06) nor for telephone consultations for all diagnoses for list-holding GPs (IRR 1.02, 95% CI 0.97–1.07) or out-of-hours GP cooperatives (IRR 0.97, 95% CI 0.86–1.10).

**Fig. 6:**
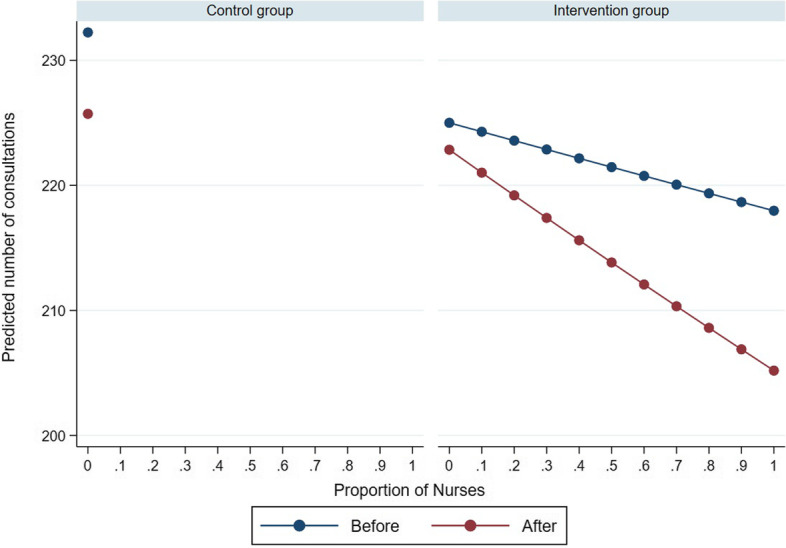
Predicted number of RTI-consultations in the out-of-hours GP cooperatives as a function of the number of nurses per 1000 inhabitants who started or completed the e-learning course per study time and group

The three-way interaction effect that could have explained how the slope of the number of RTI-consultations varies as a function of nurses per 1000 inhabitants who attended the e-learning course per study time and study group could not be estimated, as illustrated in the margins plot in Fig. 6.

## Discussion

The patient population attending the out-of-hours service during the winter season was younger and had a higher proportion of RTIs than the population attending the regular GP offices. The educational intervention about RTIs and communication skills for telephone triage nurses did not decrease the number of consultations for RTIs in the out-of-hours GP cooperatives.

### Population and epidemiology at baseline

The out-of-hours patient population is characterized by a higher proportion of children and teenagers compared with list-holding GPs’ patient population, especially for RTIs. Danish parents reported a perceived need for prompt action, unpleasant symptoms and worry as some of the main reasons for contacting the out-of-hours service [31]. This is coherent with a high rate of out-of-hours consultations for the youngest children, because they often become abruptly ill with unpleasant symptoms. Nevertheless, generally harmless conditions and insecurity concerning the assessment of respiratory symptoms of their own children may lead parents to call the out-of-hours service. Telephone triage nurses describe this group of callers as challenging, and the parents’ high degree of worry will often overrule the nurses’ medical assessment, contributing to a high consultation rate for children [15].

The proportion of sore throat diagnosed in the out-of-hours cooperatives is high. In our data, sore throat is consistent with suspected or detected streptococcal tonsillitis. There is a strong Norwegian tradition of performing rapid strep A test and initiating treatment with penicillin V if streptococci are confirmed. This tradition may contribute to high expectations in the population of a doctors’ assessment for sore throat, leading to high out-of-hours consultation rates [15].

Acute laryngitis is a hyperacute condition and tends to arise during night-time. Therefore, the high proportion of patients being diagnosed with this condition out-of-hours is unsurprising. However, the proportion of sinusitis cases was low. This finding is consistent with the low-urgency nature of sinusitis and the existing guidelines recommending a wait-and-watch attitude for this condition [32].

### Effect of the intervention

Dutch GPs believe that a stricter triage and annual feedback to triage nurses will reduce the number of non-urgent visits to the out-of-hours cooperatives [8]. This viewpoint is supported by a Finnish training intervention among nurses in an emergency unit that improved the quality of telephone triage as measured using self-administered questionnaires [33]. Self-reported increase in quality as measured using questionnaires may be an indication of quality improvement, but the lacking patient outcome is a major weakness of this and other studies on educational interventions for telephone triage nurses [28]. The present study meets the need for data on patient outcome. However, the intervention would have benefited from a process analysis to evaluate whether it made the nurses more confident in assessing callers with symptoms of mild-to-moderate RTIs.

The decision to seek health care is a complex process influenced by several factors, such as personal, social, and cultural, as well as characteristics of the health care system [4, 34]. An introduction of school requirement for a sick leave certificate for teenagers led to increased list-holding GP attendance and may contribute to out-of-hours health seeking behaviour [35]. Convenience considerations and GPs’ low capacity for urgent, same-day consultations may be other reasons for seeking help out-of-hours [31, 36, 37]. Moreover, social network is also an important determinant for help seeking behaviour regarding antibiotic use [38]. Telephone triage nurses described the fee-for-service plan for GPs working in the out-of-hours cooperatives as a perverse incentive, pushing non-urgent consultations out-of-hours [15].

Hence, there may be several reasons for the lacking effect of our educational intervention on the primary and secondary outcomes. Strategies aimed at reducing out-of-hours attendance by improving the skills of individual nurses imply that they perform an active gatekeeping role, keeping low-urgency patients out of the cooperatives. From this perspective, an educational intervention similar to the one described in the present study could be expected to have a direct effect on the out-of-hours attendance, particularly because nurses report that the decision regarding the need for a doctor’s consultation is generally made in co-operation between the nurse and caller [15].

However, our e-learning course and group discussion of 90 minutes each may be too limited to oust the external factors telephone triage nurses describe as decisive and non-controllable [15]. This point of view is supported by a Swedish study that concluded that multiple organizational factors, including the triage performed and the self-care advice provided by the nurses, play an important role in primary health care centres where antibiotic prescribing is low [39]. Hence, keeping non-urgent patients from doctors’ consultations out-of-hours is not a simple decision for one particular nurse. As for the problem of crowding in emergency departments in hospitals, the challenge might be better addressed through system changes [40].

### Methodological considerations

The data from the Norwegian Directory of Health are reliable and a good source of information about the study population as well as activity in primary health care [37]. Our study covers almost 60% of the Norwegian population, indicating high external validity in health care systems similar to the Norwegian health care system. We consider the data to be a reliable source of information about the epidemiology of RTIs during the two winter seasons. The first Norwegian case of coronavirus disease 2019 appeared on 26 February 2020, i.e., towards the end of the intervention period [41]. Hence, the effect of the coronavirus disease 2019 pandemic on the data is limited.

The large proportion of the population being covered by the included out-of-hours GP cooperatives, in addition to narrow confidence intervals, indicate that the present study has statistical power to answer our research question.

The e-learning course encouraged the nurses to not overrule the triage system in use in their own out-of-hours GP cooperative. Because many triage systems are less specific with regard to RTIs, generally with high sensitivity and low specificity for serious disease, this may have been an obstacle for change [42]. In this way, the e-learning course may have contributed to the nurses feeling more confident, without leading to a decrease in the number of callers being assigned a doctor’s consultation.

The pragmatic intention-to-treat design yielded limited control on the implementation of the intervention. We do not know how many nurses completed the two educational parts, and how many nurses had access to the course without attending it. This makes the assessment of the effect of the intervention more uncertain. To compensate for this weakness, we included the number of nurses who had started on or completed the e-learning course per 1000 inhabitants as a variable in the regression model. This variable did not have a significant effect (IRR 0.98, 95% CI 0.80–1.19). The sensitivity analysis of this variable was not possible to perform, since the nurses in the control group were not exposed and the proportion of nurses in this group is zero.

A longer intervention period might have increased the likelihood of a higher participation rate among nurses, but it would also increase the risk of external bias from the coronavirus disease 2019 pandemic. Hence, it is difficult to conclude about a lacking effect of the intervention based only on the primary and secondary outcomes. A pragmatic design is closer to real life and healthcare. We therefore considered it a pertinent method.

## Conclusion

The described educational intervention for telephone triage nurses did not influence the out-of-hours attendance for RTIs or list-holding GPs’ attendance. Changing a population’s health service attendance is complicated, and the intervention’s pragmatic design with a lack of control on the proportion of participating nurses, and its limited scope, may be reasons for the missing effect on the primary and secondary outcomes. Future studies on educational interventions should include a process evaluation, be planned for a longer period, and involve both health planning councils and leaders at different levels, as well as GPs and nurses in out-of-hours cooperatives. Furthermore, a need exists to explore the perspective of callers with RTIs who are advised to wait-and-watch or seek their list-holding GPs to reduce non-urgent use of the out-of-hours service.

## Supplementary Information

**Additional file 1.** Appendix A

**Additional file 2.** Appendix B

**Additional file 3.** Appendix C

## Data Availability

The raw data analysed during the current study contains person sensitive information and publication of these data has not been approved by The Norwegian Data Protection Authority. However, a modified dataset is available from the corresponding author on reasonable request.

## Abbreviations

CI: Confidence interval
GP: General practitioner
IRR: Incidence rate ratio
RTIs: Respiratory tract infections

## Footnotes

1 Lindberg BH, Høye S. Own report. [Raw data fetched from the KUHR-database 19.05.2019 at [https://opnedata.helserefusjon.no/]. Oslo: Helsedirektoratet [The Norwegian Directorate of Health]; 2019.
