# Supplementary material for: Effect of an educational intervention for telephone triage nurses on out-of-hours attendance: a pragmatic randomized controlled study
Source: BMC Health Serv Res. 2023 Jan 3;23:4. doi: 10.1186/s12913-022-08994-0 (PMC9807970; doi:10.1186/s12913-022-08994-0)
Supplement: Supplementary file 1 — Additional file 1. Appendix A [file 12913_2022_8994_MOESM1_ESM.docx]

# Appendix A

Headings and themes of the e-learning course of about 90 minutes:

- How the population uses out-of-hours services
  - RTIs in GP offices
  - RTIs in out-of-hours GP cooperatives
  - The effect of antibiotics
- About respiratory tract infections
  - Limitations of triage tools
  - Anatomy of the respiratory tract
  - Signs of RTIs
  - Self-limiting RTIs
  - When are RTIs high-urgency cases?
- Emergency medicine versus infectious medicine
  - Fever in adults and children
  - General condition
- Possible course of action for respiratory tract infections
  - Time as the most important curative factor
  - Antibiotics and unwanted effects
  - Prescription free drugs
- The role of telephone triage nurses
  - The many tasks of a nurse
  - The right level of care
  - The toolbox of measures
  - Busyness
- To clarify the reason for encounter
  - Different approaches
  - Patient centred approach
- Communication and negotiations
  - The significance of patient centred communication
  - Professionalism and assessments
- How to make security nests
  - About security nesting
  - Difficult conversations

The e-learning course was interactive and based on clinical cases.
