# Supplementary material for: Effect of an educational intervention for telephone triage nurses on out-of-hours attendance: a pragmatic randomized controlled study
Source: BMC Health Serv Res. 2023 Jan 3;23:4. doi: 10.1186/s12913-022-08994-0 (PMC9807970; doi:10.1186/s12913-022-08994-0)

A translated version of the template guide:

# Respiratory tract infections in the out-of-hours GP cooperative

## -Input for the completion of part two of the course

You are now in the process of completing part two of the course on respiratory infections. This part of the course is at least as important as part one (the e-learning course), and it is a great advantage if everyone has completed the e-learning course before part two is completed.

We hope that the report sent to you will help you have a good, professional conversation about your own out-of-hours GP cooperative. The intention is not for you as a group leader to be the expert in the discussion, but that you should facilitate good group dynamics and make sure that everyone makes oneself heard.

We recommend that you spend 2x45 minutes on the group conversation, but it can also work with 30 minutes if you don't have more time at your disposal.

We have created a PowerPoint presentation that you can use as an introduction to your team meeting if you have the time and if you want. There are notes for each slide. Your notes are important for reviewing your presentation. Where there are multiple points on the slide, your notes are organized so that the number on the slide has a corresponding number in your notes. We have created a separate pdf file where there is one page per slide, and where the notes belonging to the individual slide are below this. Feel free to run through your PP presentation a few times beforehand to get to know the animation and notes that come with it.

Here's our suggested program for the group meeting:

1. Show the PowerPoint presentation.
2. Quick talk in the group about the e-learning course (here you as the leader of the group discussion can choose what you want to talk about if you have limitedtime at your disposal)

- What is the impression after completing the e-learning course?
- What was new?
- Is something unclear or is there something you disagree with?
- Has the course provided new tools for communicating with callers?
- Has anyone tried new communication tools after the course?
- Anyone who wants to share their experiences with the group?

1. The report is handed out. It is important that you wait until you have finished your conversation about the e-learning course. There is input for discussion under each chapter. These are intended as a starting point. It is more important to have a good professional conversation about the use of the out-of-hours GP cooperative for respiratory infections than to get through all the points. At the same time, we recommend having plenty of time for Chapter 5, which deals with diagnoses set after telephone consultations.
2. Summarizing conversation about the way forward. Discussion input:

- Should anything be changed at your out-of-hours GP cooperative?
- If so, how should it be done?
- Who takes responsibility for implementing any changes?
- When and how should any new courses of action be evaluated?

If you have a short time at your disposal, we recommend that you only spend 2-3 minutes at point 1, and that the rest of the time is spent on points 3 and 4.

The aim of this course has been for out-of-hours nurses to gain more knowledge about respiratory tract infections and communication, so that they feel safer when they assess callers with respiratory tract symptoms. The intention is not to prevent all these callers from getting an appointment in the out-of-hours GP cooperative, but that the nurses are more able to reach agreement with the caller on courses of action other than out-of-hours consultations for mild to moderate respiratory tract infections. It has also been a goal that the nurses should be even more confident of symptoms that indicate a serious course of respiratory infection, so that these can be identified and quickly get an appointment in the out-of-hours GP cooperative.

Feel free to contact Bent Lindberg for questions, input, or views, both before, during and after the completion of the course.

E-post: [b.h.lindberg@medisin.uio.no](mailto:b.h.lindberg@medisin.uio.no)

Phone: 40220320

Good luck completing the course!

Sincerely,

Bent H. Lindberg Ingrid K. Rebnord Sigurd Høye

ASP/UiO Norce/UiB ASP/UiO


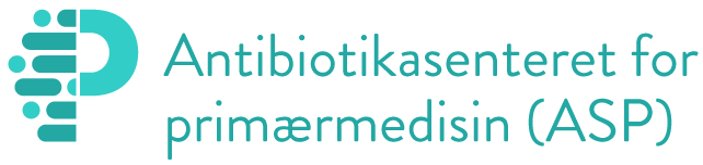

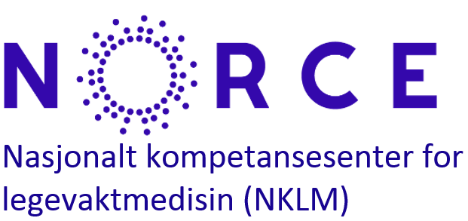

Supplement: Supplementary file 2 — Additional file 2. Appendix B [file 12913_2022_8994_MOESM2_ESM.docx]
