# Supplementary material for: Effect of an educational intervention for telephone triage nurses on out-of-hours attendance: a pragmatic randomized controlled study
Source: BMC Health Serv Res. 2023 Jan 3;23:4. doi: 10.1186/s12913-022-08994-0 (PMC9807970; doi:10.1186/s12913-022-08994-0)
Supplement: Supplementary file 3 — Additional file 3. Appendix C [file 12913_2022_8994_MOESM3_ESM.docx]

**Respiratory tract infections in the out-of-hours service – report for the year 2018**

Consultations with list-holding GPs and out-of-hours in XXX out-of-hours GP cooperative’s area


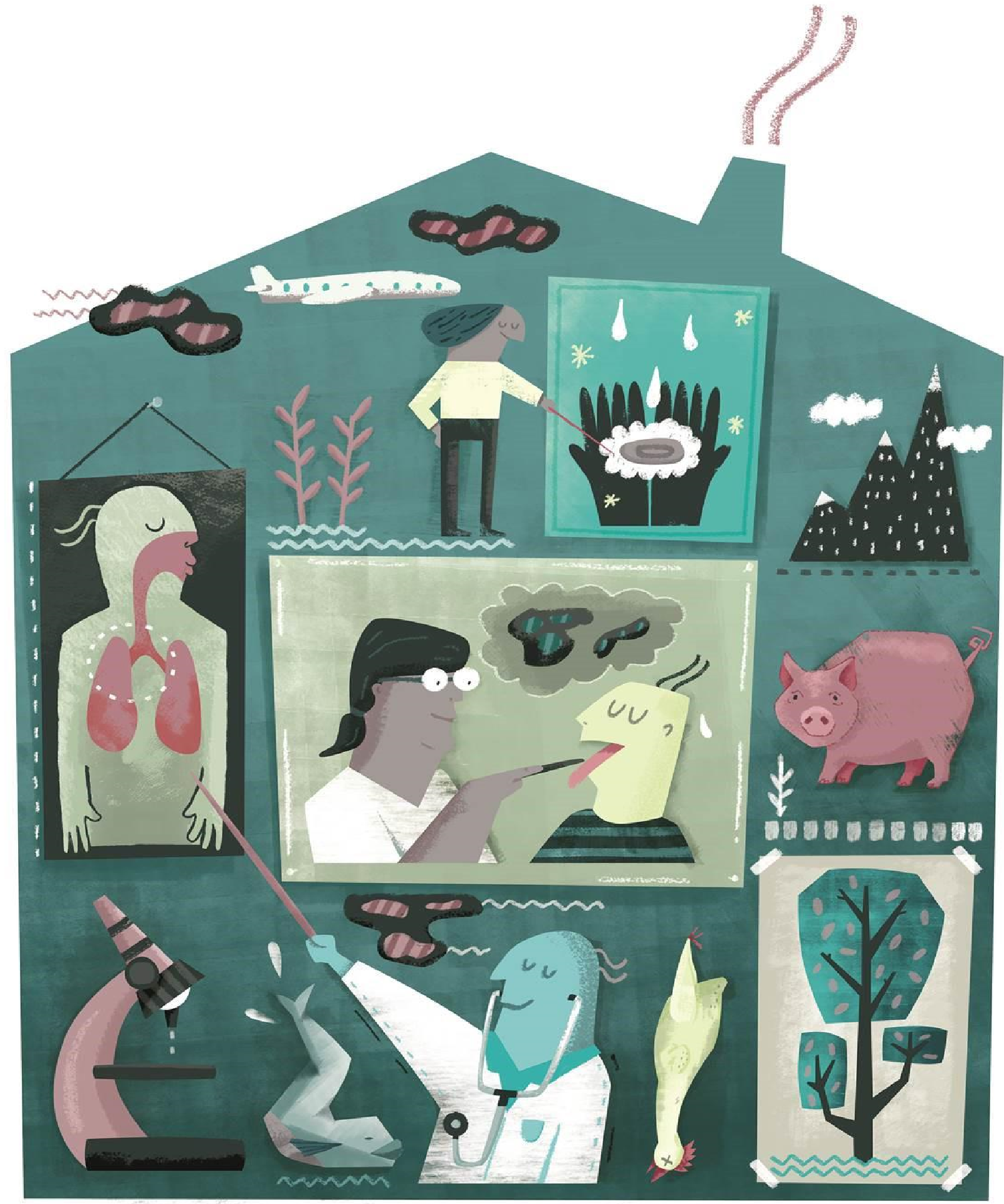

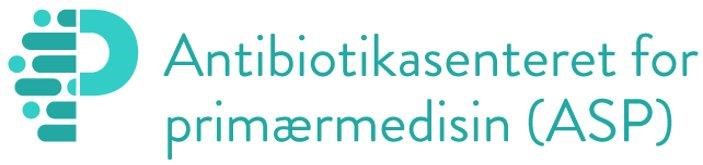

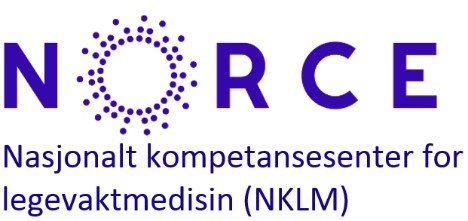


**About the report**

In this report, we show how the out-of-hours medical service and the GP service are used in your out-of-hours care area, compared to the whole country. The report shows figures for the year 2018.

We show both total figures for all diagnoses, and more specifically for respiratory tract infections. We also show figures for different age groups and for different contact types (i.e. consultations/home visits and counselling by telephone).

The purpose of this report is to initiate a conversation about how your out-of-hours GP cooperative is used by the population of the area. Of course, there is no answer as to how out-of-hours and GP services should be used, and there can be many reasons why the use in your area is different from its use in the rest of the country. Perhaps the report could still lead you to find out that you will try to bring about a change. If so, it is important to discuss measures that can bring the desired change.

The report is based on data from the KPR-register. All the data we have used are publicly available: https://helsedirektoratet.no/statistikk-oganalyse/statistikk-fra-kommunalt-pasient-og-brukerregister-kpr The data is collected from the electronic compensation claims submitted from GPs, out-of-hours doctors and out-of-hours GP cooperatives. Although there will always be some errors in such registry data, KPR data is considered good. We therefore believe the report is trustworthy. However, there may be systematic differences between different out-of-hours GP cooperatives when it comes to submitting electronic compensation claims. For example, some cooperatives may routinely charge 1bd ("Simple patient contact by paper letter or phone call") every time the nurse gives advice by phone, while other cooperatives do not have such a routine. This will of course be reflected in the report.

Respiratory tract infections is a common term for infections of the nose, pharynx, larynx, trachea, bronchi and lungs. In primary care, one works in different conditions than in hospitals, and the diagnosis itself can be somewhat more uncertain and inaccurate. When using symptom diagnoses (e.g., R05 Cough), it may reflect that one is unsure what kind of infection causes the symptom in question. However, it is always a goal to make the most accurate diagnosis (e.g., R78 bronchitis). In the report, we have retrieved data for the following diagnoses:

Ear infection: H01 ear pain, H71 acute middle ear infection, H72 serous inflammation

Respiratory symptoms: R01 to R29 (except R06 nosebleed)

Tonsillitis: R72 streptococcal tonsillitis and R76 acute tonsillitis

Colds: R74

Sinusitis: R75

Bronchitis: R78

The flu: R80

Pneumonia: R81

Other lower respiratory tract infections: R71, R82 and R83

# Key figures

This report describes **XXX out-of-hours GP cooperative**

Number of inhabitants in the area: 83488

Number of inhabitants in Norway as of 1 January 2018: **5 328 212**

Your area includes 1.58% of the country's population.

# Description of your area

There may be slightly different age distributions in different parts of the country. For example, if there are many children and adolescents in your area, this may result in a slightly different use of the out-of-hours GP cooperative. Here we show how the age distribution in your area compared to the whole country. The lines describe how many percent of the population is 0-10 years old, 11-20 years, etc.

%

0

2

%

4

%

6

%

8

%

%

10

12

%

%

14

%

16

0-10

year

11-20

year

21-30

year

31-40

year

41-50

year

51-60

year

61-70

year

71-80

year

Over 80 years

Entire Norway

Your area

***Discussion input:***

Note the age distribution in your area compared to the rest of the country. How does it affect the working conditions in your out-of-hours GP cooperative?

# Use of out-of-hours GP cooperative (all diagnoses)

How the population uses out-of-hours GP cooperatives and list-holding GPs varies slightly from area to area. There can be many factors that contribute to such differences, such as stability in the GP service, geographical distance to the out-of-hours GP cooperative's location or established routines for cooperation between GPs and the out-of-hours service. In the figures below, we have only commented on the figures that apply to the whole country.

## Total number of out-of-hours consultations per 1 000 inhabitants

| \|  \|  \|  \|  \| \|  \|  \|  \|  \|  \| \| --- \| --- \| --- \| --- \| --- \| --- \| --- \| --- \| --- \| --- \| \|  \|  \|  \|  \| \|  \|  \|  \|  \|  \| \|  \|  \|  \|  \|  \|  \|  \|  \|  \|  \| \|  \|  \|  \|  \|   200 205 210 215 220 225 230 235 240 245  Number of out-of-hours consultations per 1 000 inhabitants  Entire Norway Your Area | 250 |
| --- | --- | --- | --- | --- | --- | --- | --- | --- | --- | --- | --- | --- | --- | --- | --- | --- | --- | --- | --- | --- | --- | --- | --- | --- | --- | --- | --- | --- | --- | --- | --- | --- | --- | --- | --- |

The figure shows how many **out-of-hours** consultations and home visits per 1000 inhabitants there were in the period in your area compared to the rest of the country. In Norway, there were 245 out-of-hours consultations per 1,000 inhabitants, i.e., around 1 in 10 residents visited the out-of-hours service during the period.

## Distribution of GP consultations and out-of-hours consultations all diagnoses

0

%

%

10

%

20

%

30

40

%

50

%

60

%

70

%

80

%

90

%

100

%

Your area

Entire Norway

Percentage of GP

-

and out-of-hours consultations, all diagnoses

OOH

List-holding GP

The figure shows the share of all GP consultations and home visits in either the out-of-hours service or list-holding GPs for all diagnoses.

***Discussion input:***

How is the collaboration between GPs and the out-of-hours service in your area?

How is the allocation of work between GPs and the out-of-hours service in their area?

# Use of out-of-hours GP cooperative (respiratory tract infections)

Compared to other diagnoses, respiratory trat infections are twice as common in out-of-hours care as in GP practices. The reasons why respiratory tract infections are a typical out-of-hours problem, may be that they are acute conditions that occur rapidly, and that the ailments often worsen in the evening and in case of flat rent. In addition, many are concerned about a serious course of the disease. Nevertheless, there is some variation between different areas regarding how out-of-hours services and GPs are used for respiratory tract infections.

**3.1 Number of out-of-hours consultations for respiratory tract infections per 1 000 inhabitants**

35

36

37

38

39

40

41

42

43

44

45

Number of out-of-hours consultations for respiratory infections per 1000 inhab.

Entire Norway

Your area

The figure shows the number of doctor consultations and home visits with a diagnosis of respiratory tract infection in **the out-of-hours GP cooperative** in the period, per 1000 inhabitants, in your area and throughout Norway. In Norway, there were 44 such consultations per 1,000 inhabitants.

In your district, there were 38 out-of-hours consultations per 1,000 inhabitants.

**3.2 Distribution of GP consultations and out-of-hours consultations for respiratory tract infections**

0

%

10

%

%

20

%

30

40

%

50

%

60

%

70

%

80

%

90

%

100

%

Your area

Entire Norway

Share of GP

and out-of-hours consultations, respiratory tract infections

OOH

List-holding GPs

The figure shows the share of all respiratory tract infection consultations (including home visits) that take place in either the out-of-hours GP cooperative or with the list-holding GPs. In Norway, 14% of all these consultations take place out-of-hour, while 86% take place in GP practice. What's it like in your area?

**3.3 Distribution of respiratory tract infection consultations and all other consultations in out-of-hours and GP practice**

The figure shows how common respiratory infections are in the out-of-hours service and in GP practice. In all Norway, 18% of all out-of-hours consultations are due to respiratory infection. This means that around 1 in 6 patients in the out-of-hours service are due to respiratory tract infections. In GP practice, around 1 in 10 patients come due to respiratory tract infections. What's it like in your area?

**3.4 Various respiratory tract infections, your area**


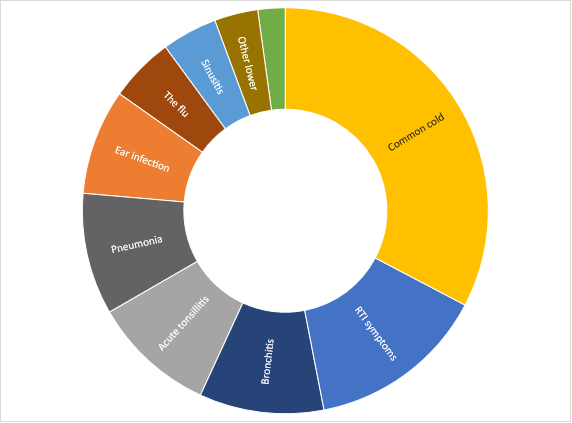


The figure shows the proportion of the various respiratory tract infections diagnosed in a doctor's consultation at your out-of-hours GP cooperative during the period.

**3.5 Various respiratory tract infections compared to GP practices and the whole country**

The figure shows the proportion of the various respiratory tract infections diagnosed in doctor consultation and home visits in your area and throughout the country, for both out-of-hours services and GP practice during the period. The figures for your out-of-hours cooperative are the same as in Figure 3.4. In Norway, common cold and the collective group "Respiratory symptoms" are the most common diagnoses to be made in case of respiratory tract infections in the out-of-hours service. What's it like in your area?

***Discussion input:***

How does the population in your area use the out-of-hours GP cooperative for respiratory tract infections?

How does the distribution of diagnoses in your out-of-hours GP cooperative compared to the rest of the country? How do you experience the collaboration with GPs around this group of patients?

# Age distribution

In this section, we will look at how different age groups use out-of-hours care and list-holding GPs. It may be useful to look at the figures in light of the figure in Chapter 1, which shows the age distribution in your area. Perhaps there are especially many young people in your area? Or especially a lot of old people?

**4.1 All diagnoses out-of-hours GP cooperatives**

%

0

%

2

4

%

%

6

%

8

10

%

%

12

14

%

16

%

%

18

%

20

0-10

year

11-20

year

21-30

year

31-40

year

41-50

year

51-60

year

61-70

year

71-80

year

Over 80 years

Entire Norway

Your area

The figure shows the share of all out-of-hours consultations for each of the age groups in your district and in the country. Throughout Norway, the share of 0- to 10-year-olds is 17% of all consultations out-of-hour. What's it like in your area?

**4.2 Respiratory tract infections in the out-of-hours service**

%

0

5

%

10

%

15

%

20

%

%

25

30

%

%

35

%

40

0-10

year

11-20

year

21-30

year

31-40

year

41-50

year

51-60

year

61-70

year

71-80

year

Over 80 years

Entire Norway

Your area

The figure shows the share of respiratory tract infections consultations out-of-hours belonging to each age group in your district and in the country.

**4.3 Respiratory tract infections in the out-of-hours service and GP practice in your area**

%

0

%

5

%

10

15

%

20

%

%

25

30

%

0-10

year

11-20

year

21-30

year

31-40

year

41-50

year

51-60

year

61-70

year

71-80

year

Over 80 years

OOH

GP

The figure shows the share of all respiratory tract infection consultations for each of the age groups in your area. The out-of-hours line is the same as in Figure 4.2, but here out-of-hours service and GP practice are compared in your area. At your out-of-hours GP cooperative, the youngest age group accounts for around 28% of all respiratory consultations.

***Discussion input:***

How would you describe the age distribution of your out-of-hours GP cooperative compared to the rest of the country?

How would you describe the age distribution of physician consultations for respiratory tract infections in your area compared to the out-of-hours service in the rest of the country? Or with GP practice in your area?

What do you think the cause of any difference could be?

# Contact types

**5.1 Number of telephone consultations in the out-of-hours service**

0

50

100

150

200

250

300

350

Number of phone numbers

-

consultations per 1000 inhabitants, in OOH

Your area

Norway

The figure shows the number of telephone consultations per 1000 inhabitants for the whole country and your district. For the whole country, there were about 120 telephone consultations per 1,000 inhabitants. This means that 1 out of 8 people had telephone consultation with the out-of-hours service. How's your district?

**5.2 Distribution of different diagnoses on telephone consultations**

How telephone consultations are used varies widely between out-of-hours GP cooperatives. In Norway, 25% of all contacts to the out-of-hours service is handled by a nurse alone. It is the diagnoses that are made in these telephone consultations that form the basis for the figures in this chapter.

0

%

10

%

%

20

30

%

40

%

50

%

60

%

70

%

80

%

90

%

100

%

Norway

Your area

share of diagnoses in telephone consultations, out-of-hours services

A99

Respiratory tract infections

All other diagnoses

The figure shows what diagnoses patients receive when consulting in the out-of-hours GP cooperative. In Norway, the unspecific diagnosis of A99 ("Health Problem/Disease") is used in 52% of telephone consultations.

In your area, the diagnosis of A99 is used in 58% of telephone consultations.

**5.3 Share of telephone consultations in total and for respiratory tract infections in the out-of-hours service**

The figure shows the share of respiratory tract infections that are handled by telephone and during consultation/home visits in your area compared to the whole country. The figure also shows how this distribution is for all diagnoses for your area and for the whole country.

***Discussion input:***

How are telephone consultations used in your out-of-hours GP cooperative?

How is the diagnostic system used when the nurse give advice solely by phone in your area?

How do you assess the relationship between telephone consultation with a nurse and consultation with a doctor for the different diagnoses?

# The way forward

The aim of this course has been for you as a nurse to gain more knowledge about respiratory tract infections, so that you feel safer when you assess callers with these infections. The intention is not to prevent all these callers from getting an appointment in the out-of-hours GP cooperative, but that you are more able to reach an agreement with the caller that a medical consultation is not always required for respiratory tract infections with mild to moderate symptoms. It has also been a goal that you should be even more confident of symptoms that indicate a serious course of respiratory tract infections, so that these can be identified and quickly get an appointment in the out-of-hours GP cooperative.

***Input to discussion after completed e-learning course and group discussion:***

What is your impression?

Do you want to implement changes? Which?

How do you want to implement these changes?

What measures will you take?

How can you measure the effect of the implemented change?
